# Distribution of γ-tubulin ring complex at the *Schizosaccharomyces pombe* spindle pole

**DOI:** 10.17912/micropub.biology.000464

**Published:** 2021-09-16

**Authors:** Sajjita Saha, Jay R Unruh, Sue L Jaspersen

**Affiliations:** 1 Stowers Institute for Medical Research; 2 Department of Molecular & Integrative Physiology, University of Kansas Medical Center

## Abstract

Microtubule nucleation is mediated by the conserved γ-tubulin ring complex (γ-TuRC). Using super-resolution microscopy, we investigate the distribution of γ-TuRC components at the spindle pole body (SPB) in wild-type *Schizosaccharomyces pombe* . We observed asymmetric distribution of γ-TuRC on its nuclear and cytoplasmic surfaces, consistent with the uneven distribution of microtubules. Examination of deletion mutants in the three non-essential γ-TuRC subunits showed defects in γ-TuRC accumulation on the old and new SPB, particularly in cells lacking *alp16+* (the Gcp6 ortholog) that may explain the monopolar spindles observed in this mutant upon mitotic entry.

**Figure 1. Distribution of γ-tubulin ring complex proteins in wild-type and mutant fission yeast cells:**
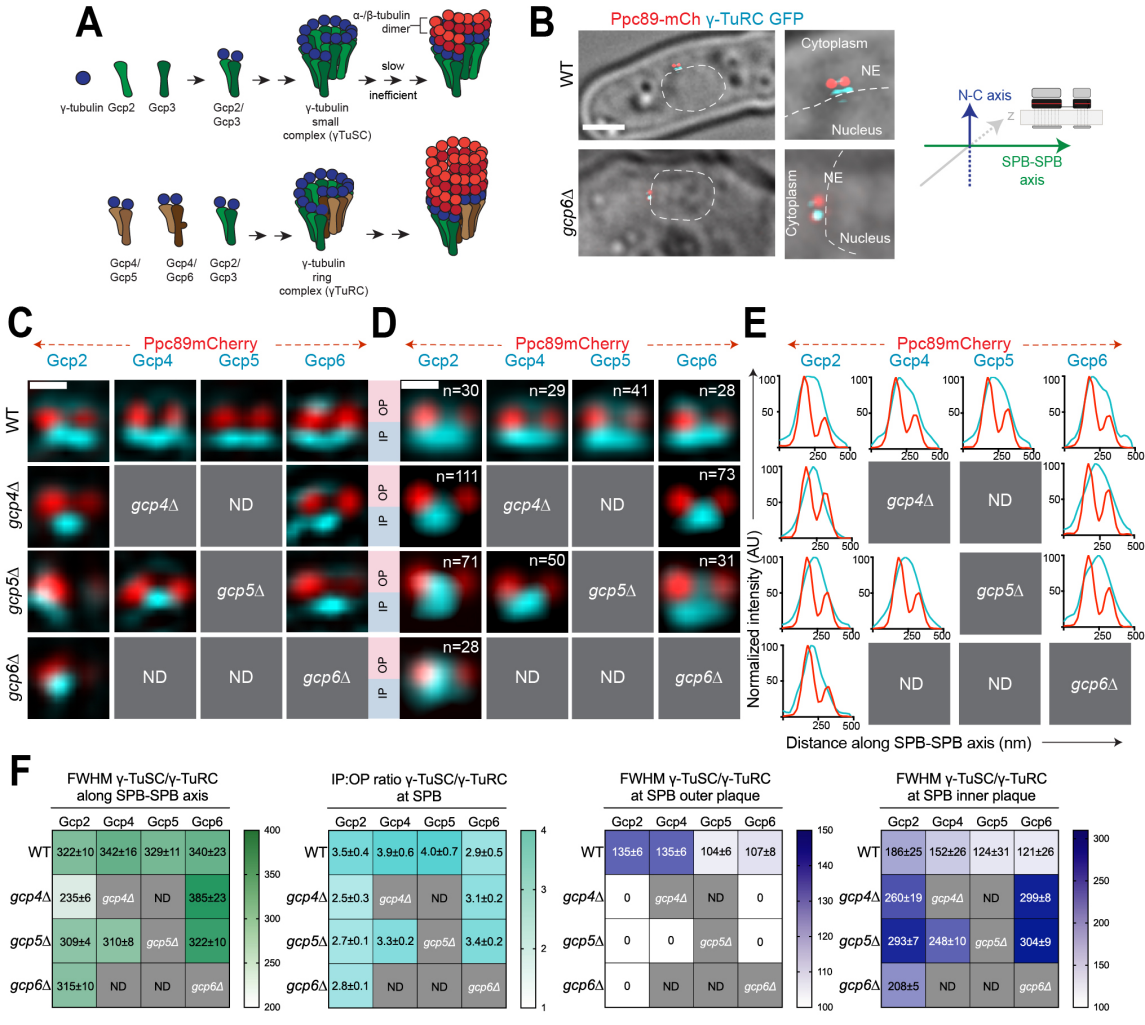
(A) Two γ-tubulin (Gcp1/Gtb1) molecules and a heterodimer of Gcp2/Alp4 and Gcp3/Alp6 form a core nucleation module known as the γ-TuSC. In fission yeast and metazoans, additional tetramers between γ-tubulin molecules and Gcp4-6 (Gfh1, Mod21 and Alp16) result in the γ-TuRC. (B-F) Structured illumination microscopy (SIM) images of wild-type (WT) and deletion strains that were GFP-tagged (cyan) at the endogenous locus for the indicated γ-TuSC (Gcp2) or γ-TuRC (Gcp4-6) protein. Ppc89-mCherry (red) in each strain marked the position of the SPB. (B) Example of a wild-type cell and *gcp6∆* mutant in which the SIM image is superimposed on the widefield image. This enabled us to determine cell cycle stage, old versus new SPB using Ppc89-mCherry intensity and nuclear versus cytoplasmic sides of the SPB using the nucleus (dashed lines) as landmarks. All images were aligned as depicted in the schematic, with Ppc89 shown in red. Bar, 2 µm. (C) Representative SIM images. (D) Single particle averaging (SPA) was used to combine the indicated (n) number of individual SIM images from G2/M cells. Bars in B-C, 200 nm. Because some γ-TuRC proteins did not localize to the SPB in certain mutants (Masuda and Toda 2016), these were not analyzed by SIM and are listed as ND, not determined. (E) Line profile from (D) to show the distribution γ-TuSC or γ-TuRC along the SPB-SPB axis relative to the SPBs (Ppc89-mCh). (F) The full-width half-maximum value (FWHM) of the distribution along the SPB-SPB axis was determined to compare distributions. In addition, line scans along the N-C axis were used to determine the ratio of γ-TuSC or γ-TuRC on the cytoplasmic (outer plaque; OP) and nuclear (inner plaque; IP) face of the SPB using averaged images. The FWHM of these fits is also shown, except in the case of the OP in mutants that we could not fit and have denoted with zeros. Errors, standard deviation.

## Description

Faithful chromosome segregation during mitosis requires the formation of the mitotic spindle. A key component of the spindle apparatus is microtubules, α- and β-tubulin dimers arranged into thirteen protofilaments. Nucleation of microtubules is catalyzed by microtubule organizing centers (MTOCs) (Moritz *et al.* 1995; Zheng *et al.* 1995). MTOCs are structurally diverse, but all are defined by enrichment of the γ-tubulin ring complex (γ-TuRC) composed of repeating tetrameric units of γ-tubulin and γ-tubulin complex proteins (GCPs) arranged in a helical fashion which mimics the microtubule geometry and serves as a template for microtubule nucleation (Figure 1A). Most organisms have five related GCPs (GCP2-6), but budding yeast lacks homologs of GCP4-6 and instead nucleates microtubules with a minimal version of the complex known as the γ-tubulin small complex (γ-TuSC) (reviewed in (Kollman *et al.* 2011; Lin *et al.* 2015; Liu *et al.* 2021; Teixido-Travesa *et al.* 2012)). Although the fission yeast *Schizosaccharomyces pombe* contains genes encoding all five GCPs ( *GCP2/alp4+* , *GCP3/alp6+* , *GCP4/gfh1+* , *GCP5/mod21+* and *GCP6/alp16+* ) in addition to γ-tubulin ( *GCP1* / *gtb1+* ), only the genes encoding subunits corresponding to the γ-TuSC are essential for mitotic growth (Anders *et al.* 2006; Fujita *et al.* 2002; Horio *et al.* 1991; Vardy and Toda 2000; Venkatram *et al.* 2004). If the γ-TuSC proteins are sufficient for microtubule nucleation, what is the function of the γ-TuRC? Structural analysis of γ-TuRC suggests that it is a better match for microtubule geometry; biochemical studies confirmed the γ-TuRC has an enhanced nucleation activity compared to the γ-TuSC (Erlemann *et al.* 2012; Oegema *et al.* 1999; Stearns *et al.* 1991; Vardy and Toda 2000). In addition, there is also evidence for differential localization of γ-TuSC and γ-TuRC complexes (Gao *et al.* 2019). The combined effect could translate into molecularly distinct MTOCs with differential microtubule nucleation potential, leading to unique microtubule arrays in different regions of a cell.

In yeast, the nuclear envelope (NE) remains intact during mitosis and the MTOC (known as the spindle pole body, SPB) is embedded in the membrane during all or part of the cell cycle so that it can nucleate both nuclear microtubules involved in chromosome segregation and cytoplasmic microtubules needed for spindle positioning. Most yeast, including filamentous fungi, budding and fission yeast have an increased number of nuclear microtubules compared to cytoplasmic microtubules (reviewed in (Jaspersen 2021)). Analysis of γ-TuSC and γ-TuRC distribution at the SPB of the filamentous fungus *Aspergillus nidulans* showed that γ-TuRC is recruited to the SPB nuclear surface while γ-TuSC binds to the cytoplasmic side (Gao *et al.* 2019). This differential localization is mediated by the γ-TuRC activating subunit, MztA (the ortholog of MOZART1/ *mzt1+* ) (Gao *et al.* 2019). The presence of distinct forms of microtubule nucleators on the nuclear and cytoplasmic sides of the SPB provides an example illustrating how microtubule nucleation may be controlled by γ-TuSC and γ-TuRC to result in a greater number of nuclear microtubules compared to cytoplasmic microtubules. Analysis of Mzt1 in fission yeast suggests that it plays an essential role in mitotic spindle formation likely through its role in stabilization of Gcp3 within the γ-TuSC (Leong *et al.* 2019; Masuda and Toda 2016). However, it is unknown if the distribution of γ-TuSC and γ-TuRC is regulated at the SPB in fission yeast as it is in *A. nidulans* .

To test if differential localization of γ-TuSC and γ-TuRC is a ubiquitous method used by yeast to regulate the number of nuclear and cytoplasmic microtubules, we used structured illumination microscopy (SIM) to examine SPBs in asynchronously growing *S. pombe* strains containing GFP-tagged versions of Gcp2 (γ-TuSC), Gcp4, Gcp5 or Gcp6 (γ-TuRC); Ppc89-mCherry marked the SPB core. SIM improves resolution roughly two-fold over widefield and confocal imaging methods, and it has previously allowed us to visualize the distribution of the γ-TuSC and Mzt1 in asynchronously growing cells on the nuclear and cytoplasmic facing sides of the SPB (Bestul *et al.* 2017). Under all conditions tested, Gcp2 and Gcp3 behaved identically (Bestul *et al.* 2017). Similar results were also obtained in work examining Gcp2/Gcp3 localization and binding (Anders *et al.* 2006; Leong *et al.* 2019). Therefore, we used Gcp2-GFP as a proxy for the γ-TuSC in this study. The intensity of Ppc89-mCherry was used to determine the old SPB from the pre-existing cell cycle and the newly formed SPB, while cell morphology was used to determine cell cycle position and to determine the nuclear and cytoplasmic orientation of the SPBs (Figure 1B). In G2 phase cells (9.5-11 µm with SPBs ~200 nm apart), both Mzt1 and the γ-TuSC proteins Gcp2-GFP and Gcp3-GFP accumulate strongly at the nuclear facing side of the old and new SPB with some additional material on the bridge that connects the two SPBs (Figure 1C) (Bestul *et al.* 2017). The γ-TuRC components in wild-type cells mirrored that of the γ-TuSC (Figure 1C), suggesting that the γ-TuRC is present at both the old and new SPBs.

To quantitate the relative levels of γ-TuSC and γ-TuRC at the two SPBs, we performed single particle averaging of SIM images (SPA-SIM). Using Ppc89-mCherry as a fiducial marker, this method allows us to compare distribution over multiple images, increasing resolution and eliminating artifacts that are frequently observed in SIM images (see Bestul *et al.* 2017; Burns *et al.* 2015). This analysis showed that both the γ-TuSC and γ-TuRC localize over a wide area spanning both the old and new SPBs in wild-type cells, particularly on the nuclear side of the SPB along the SPB-SPB axis (Figure 1D-E). In cells lacking *gcp4+* , the distribution of γ-TuSC along the SPB-SPB axis was reduced, based on analysis of individual and merged images and measurement of the full-width half maximum (FWHM) values of protein distribution (Figure 1D-F). Both Gcp2-GFP and Gcp6-GFP were enriched in the bridge area between the two SPBs in most *gcp4∆* mutant cells compared to the distribution to both SPBs in wild-type cells. Loss of *gcp5+* did not have a statistically significant effect on the spread of γ-TuSC and γ-TuRC along the SPB-SPB axis based on FWHM values (Figure 1F). In *gcp6∆* mutants, the spread of Gcp2-GFP along the SPB-SPB axis wa similar to wild-type cells (Figure 1F), however, inspection of individual and averaged images revealed an important difference in the placement of Gcp2. Wild-type cells had Gcp2-GFP spread between both SPBs whereas Gcp2-GFP was concentrated at the old SPB in *gcp6∆* mutants (Figure 1C-E). This observation could explain why *gcp6∆* cells, but not *gcp4∆* or *gcp5∆* mutants, give rise to monopolar spindles in prometaphase (Masuda and Toda 2016). At a mechanistic level, it is unknown why γ-TuSC is maintained at the old SPB in cells lacking *gcp6+* function but this might reflect a role for Gcp6 or Mzt1 in recruitment or stabilization of the γ-TuSC; previous work showed that Gcp6 plays a synergistic role with Mzt1 in early spindle assembly (Masuda and Toda 2016).

A comparison of γ-TuSC and γ-TuRC levels at the nuclear and cytoplasmic facing sides of the SPB, known as the inner (IP) and outer (OP) plaques respectively, showed an asymmetric distribution similar to that of microtubules themselves. Although we observed some variation in levels of individual components, most were present in 2.5-4 fold greater levels on the nuclear face compared to the cytoplasmic side in wild-type cells (Figure 1F). Based on EM analysis of microtubule distribution in fission yeast, approximately 12-15 microtubules form at the inner plaque compared to the 1-3 astral microtubules observed at the outer plaque (Ding *et al.* 1993). The observed ratio of 2.5-4 γ-TuRC on the nuclear side of the SPB compared to the cytoplasmic face suggests γ-TuRC distribution is a major factor controlling microtubule distribution. However, given that levels of γ-TuRC on the cytoplasmic face were higher than expected based on microtubule number, it seems likely that other factors beyond γ-TuRC abundance control the amount of microtubules. Leading candidates include SPB receptors, microtubule polymerases and perhaps modifications of the γ-TuRC that affect its function. Curiously, Gcp6-GFP had a lower IP:OP ratio than other components of the γ-TuSC or γ-TuRC in wild-type cells (2.5±0.9 versus 3.5-4) due to reduced levels of protein at the IP (Figure 1F). It is unknown if this is an artifact of tagging or fixation, or if it reflects compositional heterogeneity in γ-TuRC not previously appreciated in biochemical studies. A reduced IP:OP ratio was also observed for γ-TuSC in mutants in the γ-TuRC components due to loss of protein at the OP (Figure 1F).

The ability to visualize theγ-TuSC andγ-TuRC in vivo and to genetically manipulate the composition of complexes opens up new avenues to investigate how structural heterogeneity at an MTOC translates into differences in microtubule nucleation, stability and attachment. In *A.nidulans* , asymmetric distribution of γ-TuSC and γ-TuRC may underlie differences in microtubule number (Gao *et al.* 2019), while studies in budding yeast highlight the important role of theγ-TuSC receptors (Geymonat *et al.* 2020; Knop and Schiebel 1998). In vitro analysis of microtubule nucleation highlighted several key mechanistic differences for Mzt1 and Gcp3 in fission yeast (Leong *et al.* 2019) that will be interesting to follow-up with in vivo analyses of γ-TuSC andγ-TuRC assembly and distribution. Together, these results highlight the diverse mechanisms used by cells to ensure that microtubules are formed at the right time, in the correct place and in the proper number to form a bipolar spindle capable of chromosome segregation.

## Methods

Yeast strains : Standard methods were used for yeast growth and genetic manipulations (Moreno *et al.* 1991). Endogenously GFP-tagged versions of γ-TuSC and γ-TuRC were obtained Ken Sawin (University of Edinburgh) and were previously tested for functionality (Anders *et al.* 2006). *ppc89+* was fused to mCherry in these strains using PCR-based methods (Bahler *et al.* 1998). Deletion alleles were also obtained from Ken Sawin and were introduced into tagged strains by mating (Anders *et al.* 2006). A complete list of strains is listed below.

Freshly plated cells were grown at 25 ^°^ C in rich yeast extract media (YES5S) for ~24 h with back dilutions to ensure cells were in logarithmic phase. Cells were then fixed for 15 min in 4% paraformaldehyde (Ted Pella) in 100 mM sucrose and then washed three times in phosphate buffered saline (PBS), pH 7.4. Cells were resuspended in a small volume of PBS, applied to a clean glass slide with a No. 1.5 coverslip and imaged immediately.

Structured Illumination Microscopy, Single Particle Averaging and Analysis : Applied Precision OMX Blaze V4 (GE Healthcare) with 60X 1.42NA Olympus Plan Apo oil objective and two PCO Edge sCMOS cameras were used to capture all SIM images. To acquire GFP and mCherry tagged cell images, 488 nm and 561 nm lasers were used with a 405/488/561/640 dichroic and emission filters 504-552 nm for GFP and 590-628 nm for mCherry. Representative images are shown as maximum intensity projections and scaled 8×8 with bilinear interpolation. To perform SPA-SIM, we followed the method outlined in (Bestul *et al.* 2017) using custom written macros and plugins available at http://research.stowers.org/imagejplugins/ . Briefly, G2 phase cells with unseparated SPBs were selected from the asynchronous population using cell length from a widefield image and SPB-SPB distance using Ppc89-mCherry. SPBs were oriented along the SPB-SPB axis using Ppc89-mCherry intensity, which is greater at the old (Bestul *et al.* 2017) and along the N-C axis using nuclear position visible in the widefield image (see Figure 1B). SPB Plot profiles were created by drawing a 19-pixel width line across the SPB-SPB axis to determine intensity values in both channels. Intensity values were normalized and plotted in Graph Pad Prism.

Because inner and outer plaque distributions were not clearly resolved for many proteins, we used an integrated fit approach to calculate ratios shown in Figure 1F. Calculations were performed with custom code written in Python. Average intensity distributions along the SPB-SPB axis were created and fit to two Gaussian functions (for wild-type strains) or one Gaussian function (for mutant strains) by non-linear least squares. The algorithm used was the trust region reflective algorithm in the SciPy optimize package (Branch *et al.* 1999). The integrals of those functions were calculated for each side of the Ppc89-mCherry center position (as determined by a Gaussian fit as above) and then used to calculate the reported ratios using the following equations:


$$\mathrm{Left intensity}=\frac{A_{1}}{2}*\left[1+\mathrm{Erf}\left(\frac{x_{\mathrm{PPC}89}-x_{c1}}{\sqrt{2}\sigma_{1}}\right)\right]+\frac{A_{2}}{2}*\left[1+\mathrm{Erf}\left(\frac{x_{\mathrm{PPC}89}-x_{c2}}{\sqrt{2}\sigma_{2}}\right)\right]$$

$$\mathrm{Right intensity}=\frac{A_{1}}{2}*\left[1-\mathrm{Erf}\left(\frac{x_{\mathrm{PPC}89}-x_{c1}}{\sqrt{2}\sigma_{1}}\right)\right]+\frac{A_{2}}{2}*\left[1-\mathrm{Erf}\left(\frac{x_{\mathrm{PPC}89}-x_{c2}}{\sqrt{2}\sigma_{2}}\right)\right]$$

Here x _PPC89_ is the center position of the Ppc89-mCherry distribution and *A* , x _c_ , and σ are the amplitude, center, and standard deviation of the Gaussian fit with subscripts denoting multiple Gaussians and subscript 2 ignored for single Gaussian fits. Errors in these integrated intensities were obtained by the Monte Carlo method (Bevington and Robinson 2003). Briefly, 100 curves were simulated from the best fit profile with Gaussian random errors added with a standard deviation equivalent to the standard deviation of the original fit residuals. Those curves were fit, and integrals were calculated as described above. Errors are reported as standard deviations of those integral values. FWHM is 2.35 the standard deviation in the Gaussian fit.

## Reagents

**Table:** 

**Strains**	**Genotype**	**Source**
fySLJ232	*h+ alp4-GFP::kanMX6 ppc89-mCherry::natMX ade6-M210 his- leu1-32 ura4-D18*	Lab stock
fySLJ979	*h− alp16-GFP::kanMX6 ppc89-mCherry::natMX ade6-M210 leu1-32*	This study
fySLJ980	*h− gfh1-GFP::kanMX ppc89-mCherry::natMX ade6-M210 leu1-32 ura4-D18*	This study
fySLJ987	*h− mod21-GFP::kanMX ppc89-mCherry::natMX ade6-M210 leu1-32 ura4-D18*	This study
fySLJ1160	*Δgfh1::natMX alp4-GFP::kanMX ppc89-mCherry::hygMX*	This study
fySLJ1160	*Δgfh1::natMX alp4-GFP kanMX ppc89-mCherry::hygMX*	This study
fySLJ1161	*Δgfh1::natMX mod21-GFP kanMX ppc89-mCherry::hygMX*	This study
fySLJ1162	*Δgfh1::natMX alp16-GFP kanMX ppc89-mCherry::hygMX*	This study
fySLJ1163	*Δmod21::hygMX alp4-GFP kanMX ppc89-mCherry::natMX*	This study
fySLJ1164	*Δmod21::hygMX alp16-GFP kanMX ppc89-mCherry::natMX*	This study
fySLJ1165	*Δmod21::hygMX gfh1-GFP kanMX ppc89-mCherry::natMX*	This study
fySLJ1166	*Δalp16::hygMX alp4-GFP kanMX ppc89-mCherry::natMX*	This study
fySLJ1167	*Δalp16::hygMX gfh1-GFP kanMX ppc89-mCherry::natMX*	This study
fySLJ1168	*Δalp16::hygMX mod21-GFP kanMX ppc89-mCherry::natMX*	This study
